# The Sheared Central Venous Catheter?

**DOI:** 10.1155/2011/379827

**Published:** 2011-11-01

**Authors:** Harihar V. Hegde, Vijay G. Yaliwal, Shyamsundar K. Joshi, P. Raghavendra Rao

**Affiliations:** ^1^Department of Anaesthesiology, SDM College of Medical Sciences and Hospital, Dharwad, Karnataka 580009, India; ^2^Department of Radiodiagnosis, SDM College of Medical Sciences and Hospital, Dharwad, Karnataka 580009, India

## Abstract

A fractured central venous catheter (CVC) with embolization of the distal fragment may lead to life-threatening complications. We had inserted a right subclavian CVC in a 68-year-old female which upon a follow-up chest X-ray appeared to have been sheared. A guidewire was inserted through the CVC until the J-tip was just beyond the tip of the CVC which were then withdrawn as a single assembly. We suspected that the tip of the guidewire might have been entrapped in the opening of the middle port, which upon withdrawal of the guidewire could have led to CVC folding upon itself and shearing.

## 1. Introduction

Central venous catheter (CVC) insertion is a common procedure performed in hospitalised patients. In most institutes, a postprocedural chest radiograph is routinely obtained to confirm the position of the CVC and to rule out complications. The role of radiology in the diagnosis and management of complications related to central venous access is invaluable [1]. A fractured CVC with embolization of the distal fragment may lead to catastrophic complications. We report a case of central venous catheter which on chest X-ray appeared to have been sheared and generated sufficient doubts about its integrity. We discuss the possible mechanisms of shearing of a CVC.

## 2. Case Report

We had inserted a right subclavian CVC in a 68-year-old female with obstructive jaundice who was scheduled to undergo common bile duct exploration and removal of a stuck dormia basket following an endoscopic retrograde cholangiopancreatography. Before insertion, the CVC (7Fr, triple lumen catheter, 16 cm length, Arrow-Howes, Multi-Lumen Central Venous Catheterisation Set with Blue FlexTip Catheter, Pa, USA) and the guidewire were examined for any manufacturing defects and the CVC lumina were flushed with saline. An experienced anaesthesiologist performed the procedure under local anaesthesia in a single attempt. Anatomical land marks technique was used, and there were no obvious complications. However, a slight resistance was noted during withdrawal of the guidewire. The guidewire which was gently withdrawn and examined did not show any deformity or kinking.

**Figure 1:**
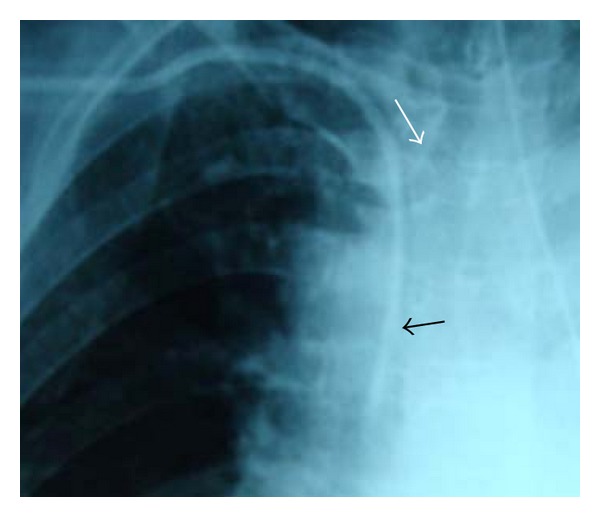
Chest X-ray showing the “sheared” (black arrow) central venous catheter and the “tracheal bronchus” (white arrow).

A follow-up chest X-ray showed a satisfactorily positioned CVC. Upon close examination, the CVC appeared to have been sheared (Figure 1), and, incidentally, the right upper lobe bronchus arising from the trachea (tracheal bronchus). We ruled out any abnormal communication between the channels of the CVC by performing a bedside test by transducing the pressure waveform via one of the ports while intravenous fluid was infused through another [2]. The radiologist opined that even though the barb appeared to be an artifact, the above possibility could not be ruled out. Possibility of a noninvasive investigative modality like a computed tomography was considered to confirm the diagnosis. However, it was opined that the diagnosis could not be confirmed/ruled out by any imaging modality. Hence, it was decided to withdraw the CVC under C-arm guidance as the patient was scheduled for the surgery. 

**Figure 2:**
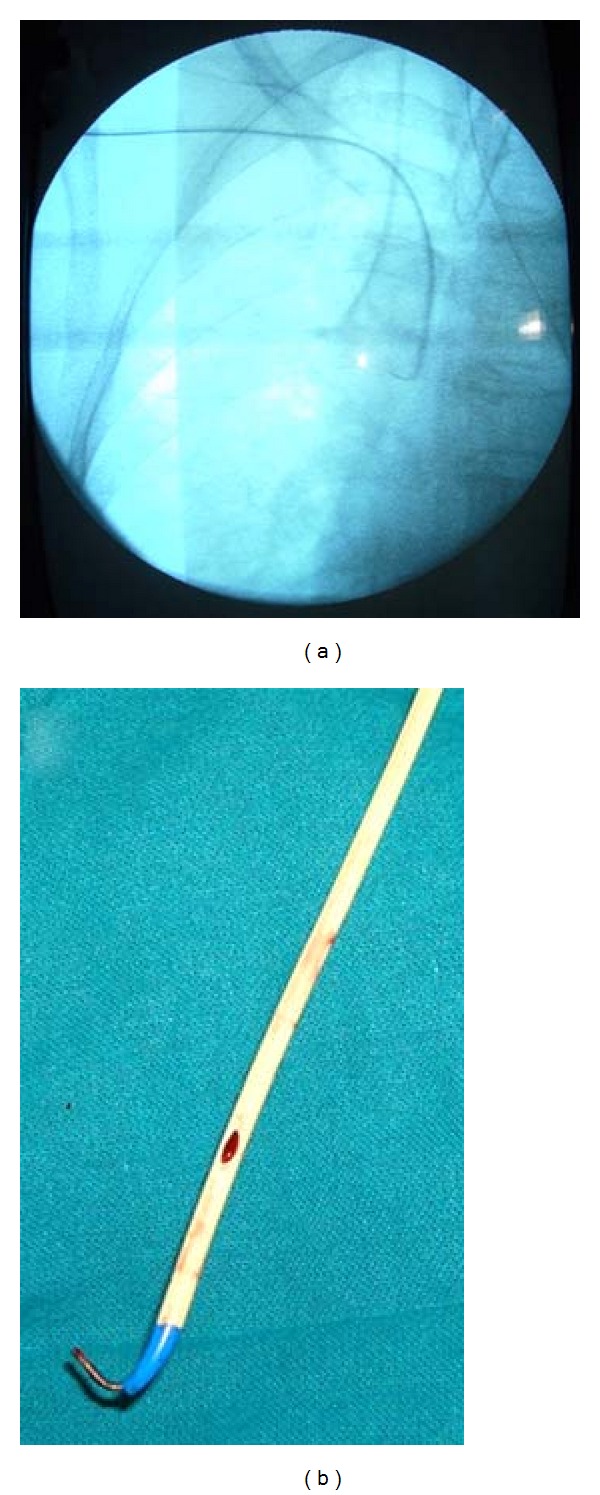
(a) Fluoroscopic image showing the right subclavian CVC with the guidewire and the ipsilateral internal jugular CVC and (b) the undamaged CVC upon removal. CVC: central venous catheter.

Two hours later, in the operation theatre, another CVC was carefully inserted through the right internal jugular vein (IJV), the position of which was confirmed by C-arm. After appropriate aseptic precautions, a guidewire was gently inserted through the right subclavian CVC (suspected to have been sheared) until the J-tip was just beyond the tip of the CVC (Figure 2(a)). The CVC and the guidewire were then carefully withdrawn as a single assembly under the C-arm guidance. Examination of the withdrawn CVC showed no abnormalities whatsoever (Figure 2(b)). 

**Figure 3:**
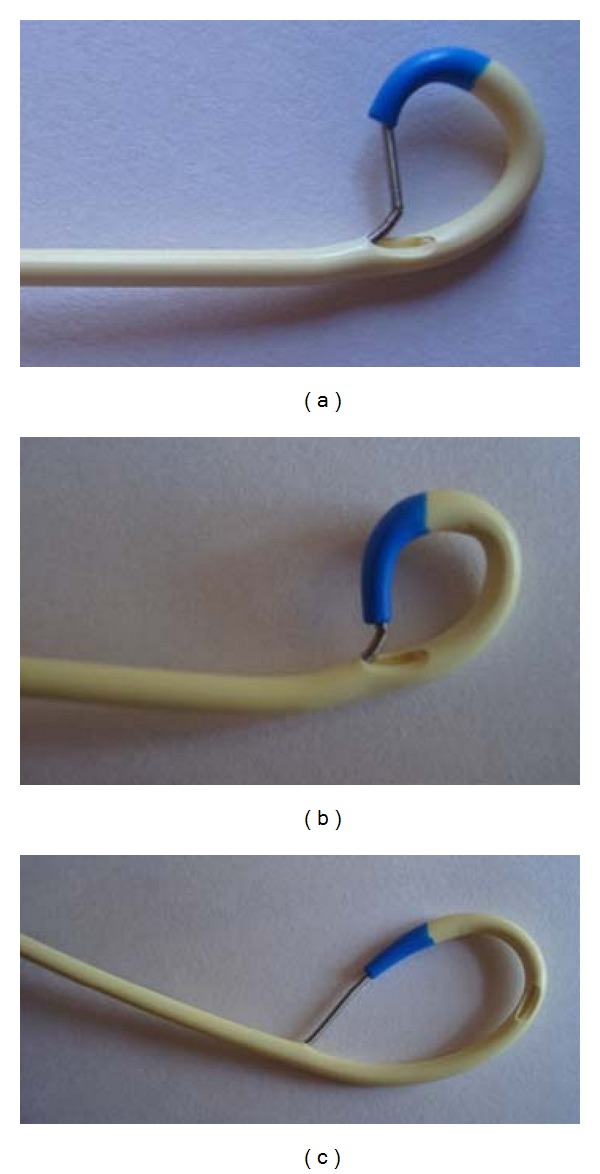
The possible mechanism of central venous catheter shear by the guidewire.

We conducted an experiment to check how possibly the CVC could have been sheared. We inserted the guidewire via the distal port of the CVC and then inserted the “J-tip” into the opening of the middle port (Figure 3(a)) and tried to pull it out through the proximal end of the distal port. We were unable to dislodge the “J-tip” from the opening of the middle port even after applying considerable traction on the guidewire, and the loop became smaller and tighter as the traction increased (Figure 3(b)). However, the CVC was not sheared even though the guidewire was damaged with application of extreme traction! We repeated the same after inserting the “J-tip” into the opening of the proximal port (Figure 3(c)). Here, the guidewire tip could be easily dislodged.

## 3. Discussion

Shearing/rupture is a recognized complication of central venous catheters with a reported incidence of 2.5% over a 5-year period at one centre. This complication is usually observed in permanent central venous access systems which are probably subjected to greater shearing between the clavicle and the first rib [3]. These catheters are also of larger diameter than those used for short term. There are early and late causes of disruption. The former appear within 24/72 hours of insertion or first use of the system and are almost always attributable to an incorrect procedure [4]. Causes that have been described include shearing by the introducer needle during insertion, high pressure within the catheter caused by bolus infusions, fracturing of the external portion by the patient's body movements (mostly infants), during removal of a stuck catheter (mostly by fibrin sheath formation around the catheter), and weakening of the catheter tip by movements of the tricuspid valve and right ventricular motion or mechanical forces between the clavicle and first rib [5].

In our case, we suspected that the “J-tip” of the guidewire might have been entrapped in the opening of the middle port (located about 2.5 cm proximal to the tip of the Arrow-Howes triple lumen catheter of 16 cm length) of the CVC (Figure 3(a)), which upon withdrawal of the guidewire could have led to the CVC folding upon itself and shearing. One would expect some deformity of the guidewire as well if this was the case. However, no such changes were observed. The chest radiograph generated sufficient doubts about the integrity of the CVC which was further supported by the circumstantial subtle increased resistance felt during the guidewire withdrawal. Under normal circumstances (without any radiological abnormality), the observations made during the CVC insertion would have been dismissed as insignificant. We considered potential consequence of a sheared CVC-like embolisation of the distal fragment (“pinch-off” syndrome) [6] and felt that removal of the CVC was the best solution as we were not sure about its integrity. Retrospectively, we conclude that the transverse process of the adjoining vertebra created an artifact which appeared like a barb on CVC. 

Our experiment reveals one thing, that is, if the guidewire tip becomes entrapped in the opening of the middle port, it is almost impossible to retrieve the guidewire. This fact poses a difficult clinical challenge as to what should be done in cases where significant resistance is noted during withdrawal of the guidewire. Based on our experiment, we suggest that no further attempt should be made to retrieve the guidewire and an immediate radiological evaluation is warranted to know the cause for such a difficulty. Such an entrapment of the guidewire may require surgical exploration for its retrieval.

Cannulation of a central vein on the same side as an existing CVC requires caution [5] as the existing CVC may be speared by the introducer needle and guidewire. Management of a fractured CVC may need assistance from interventional radiology or surgical exploration. Slow gentle traction [7] or cutaneous cutdown followed by distal venotomy [8] may be required for incomplete fractures. For complete fractures, radiological loops, snares, coils [9], or surgical exploration may be required. We are not sure whether our method of using a guidewire to support the “sheared” CVC was the best option available as the reinsertion of the guidewire itself could have caused further damage to the CVC and its rupture. However, we took enough care by being gentle during the whole exercise and performed the procedure under fluoroscopy so that any difficulty/untoward event would have been diagnosed early enough to take necessary remedial measures. We were prepared to handle the complications surgically/radiologically. In the end, the exercise was worth as it cleared the doubt once for all, even though it resulted in an additional procedure in securing another CVC and an extra expenditure in the patient care. There are several reports which describe sheared and migrated distal portion of a CVC. However, to the best of our knowledge, there are no descriptions of radiological features of a sheared CVC *in situ*, even though our report is not of a “true” sheared CVC.

Technique and the equipment-related causes for CVC shear may be preventable by careful consideration to the preuse examination of the CVC and the accessories, meticulous technique during the procedure and keeping the CVC tip in superior vena cava rather than in one of the cardiac chambers. However, little may be done to prevent patient-related factors like movement occurring between the clavicle and first rib.

Central venous catheters may be damaged any time during their usage. Early and subtle signs of a damaged CVC need to be carefully assessed and managed appropriately in view of the potential complications.
